# In Vitro Neurotoxicity of Chinese Krait (*Bungarus multicinctus*) Venom and Neutralization by Antivenoms

**DOI:** 10.3390/toxins13010049

**Published:** 2021-01-11

**Authors:** Qing Liang, Tam Minh Huynh, Yen Zhi Ng, Geoffrey K. Isbister, Wayne C. Hodgson

**Affiliations:** 1 Monash Venom Group, Department of Pharmacology, Biomedical Discovery Institute, Monash University, Clayton, VIC 3800, Australia; qing.liang@monash.edu (Q.L.); Tommy.Huynh@monash.edu (T.M.H.); yzng5@student.monash.edu (Y.Z.N.); geoff.isbister@gmail.com (G.K.I.); 2 Department of Emergency Medicine, The First Affiliated Hospital of Guangzhou Medical University, 151 Yanjiang Rd, Guangzhou 510120, China; 3 Clinical Toxicology Research Group, University of Newcastle, Newcastle, NSW 2308, Australia

**Keywords:** *Bungarus multicinctus*, neurotoxicity, envenoming, venom, antivenom, snake

## Abstract

*Bungarus multicinctus*, the Chinese krait, is a highly venomous elapid snake which causes considerable morbidity and mortality in southern China. *B. multicinctus* venom contains pre-synaptic PLA_2_ neurotoxins (i.e., β-bungarotoxins) and post-synaptic neurotoxins (i.e., α-bungarotoxins). We examined the in vitro neurotoxicity of *B. multicinctus* venom, and the efficacy of specific monovalent Chinese *B. multicinctus* antivenom, and Australian polyvalent elapid snake antivenom, against venom-induced neurotoxicity. *B. multicinctus* venom (1–10 μg/mL) abolished indirect twitches in the chick biventer cervicis nerve-muscle preparation as well as attenuating contractile responses to exogenous ACh and CCh, but not KCl. This indicates a post-synaptic neurotoxic action but myotoxicity was not evident. Given that post-synaptic α-neurotoxins have a more rapid onset than pre-synaptic neurotoxins, the activity of the latter in the whole venom will be masked. The prior addition of Chinese *B. multicinctus* antivenom (12 U/mL) or Australian polyvalent snake antivenom (15 U/mL), markedly attenuated the neurotoxic actions of *B. multicinctus* venom (3 μg/mL) and prevented the inhibition of contractile responses to ACh and CCh. The addition of *B. multicinctus* antivenom (60 U/mL), or Australian polyvalent snake antivenom (50 U/mL), at the t_90_ time point after the addition of *B. multicinctus* venom (3 μg/mL), did not restore the twitch height over 180 min. The earlier addition of *B. multicinctus* antivenom (60 U/mL), at the t_20_ or t_50_ time points, also failed to prevent the neurotoxic effects of the venom but did delay the time to abolish twitches based on a comparison of t_90_ values. Repeated washing of the preparation with physiological salt solution, commencing at the t_20_ time point, failed to reverse the neurotoxic effects of venom or delay the time to abolish twitches. This study showed that *B. multicinctus* venom displays marked in vitro neurotoxicity in a skeletal muscle preparation which is not reversed by antivenom. This does not appear to be related to antivenom efficacy, but due to the irreversible/pseudo-irreversible nature of the neurotoxins.

## 1. Introduction

*Bungarus* genus (kraits) are nocturnal venomous snakes from the Elapidae family that are only found in Asia with widespread distribution across many countries including India, Pakistan, Indonesia, Sri Lanka, Malaysia, Bangladesh, Vietnam and China. Kraits are recognized by their banded pattern and possess venoms that contain highly potent neurotoxins. Three species of *Bungarus* are found in China: *Bungarus multicinctus* (Chinese krait or many-banded krait), *Bungarus fasciatus* (banded krait) and *Bungarus bungaroides* (Himalayan krait) [1]. In China, *B. multicinctus* and *B. fasciatus* are more medically important because they are more common and, therefore, more likely to cause envenoming in humans. Although robust epidemiological data is lacking, it has been estimated that *B. multicinctus* bites account for approximately 8% of all snakebites in China, while *B. fasciatus* bites account for 2.3% [2].

*B. multicinctus* is widely distributed in southern China [1], as well as in Myanmar, north Vietnam, Laos and Thailand [3]. Although *B. multicinctus* bites are ranked as the fifth most common in China, *B. multicinctus* envenoming is ranked first for case fatality rates, ranging from 26.9–33.3% depending on geographical location [2]. The hallmark of *B. multicinctus* envenoming is progressive neuromuscular paralysis requiring prolonged artificial ventilation but with minimal local swelling and pain at the bite site [2,4,5,6]. *B. multicinctus* venom contains a large proportion of post-synaptic “three finger” toxins (3-FTx) (i.e., α-bungarotoxins) and pre-synaptic phospholipase A_2_ (PLA_2_) β-bungarotoxins [7,8]. Proteomic data indicates that PLA_2_ and 3-FTx toxins represent the most abundant components in Chinese *B. multicinctus* venom, at 66.4% and 32.6%, respectively [9]. 

Patients envenomed by *B. multicinctus* develop respiratory failure in spite of early (i.e., within 4 h of the bite) administration of the recommended quantities of *B. multicinctus* antivenom [5]. It is believed that this clinical manifestation of *B. multicinctus* envenoming (i.e., antivenom resistant neurotoxicity) is primarily due to rapid fixation and irreversible necrotic degeneration of the nerve terminal boutons by the PLA_2_ β-bungarotoxins [10,11,12,13]. Contributing to this effect, the post-synaptic neurotoxins from *B. multicinctus* venom are relatively irreversible long-chain α-neurotoxins [10,14]. However, antivenom ‘effectiveness’ is primarily evaluated in vivo by testing the ability of antivenom to prevent death in a rodent model. We have previously reported discrepancies between rodent lethality-based efficacy studies and in vitro neuromuscular preparation studies for two Malaysian Krait Species (*Bungarus candidus* and *Bungarus fasciatus*) [15].

The efficacy of Chinese *B. multicinctus* antivenom against neurotoxicity has not previously been examined in vitro. In the current study, the in vitro neurotoxic activity of Chinese *B. multicinctus* venom was assessed in the chick biventer cervicis nerve-muscle preparation. The neutralizing efficacy of the Chinese monovalent *B. multicinctus* antivenom, and the Australian polyvalent snake antivenom, against Chinese *B. multicinctus* venom was assessed. In addition, the ability of the Chinese *B. multicinctus* antivenom to reverse neurotoxicity at various time points post-venom was also evaluated.

## 2. Results

### 2.1. In Vitro Neurotoxicity

#### 2.1.1. Concentration-Dependent Inhibition of Twitches and Exogenous Agonists Responses

**Figure 1:**
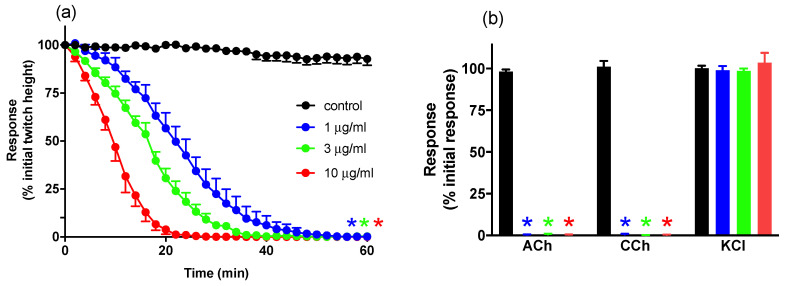
The concentration-dependent in vitro neurotoxic effects of *B. multicinctus* venom (1–10 μg/mL) on (**a**) indirect twitches and (**b**) responses to exogenous agonists acetylcholine (ACh; 1 mM), carbachol (CCh; 20 μM) and potassium chloride (KCl; 40 mM) in the chick biventer cervicis nerve-muscle preparation. * *p* < 0.05, significantly different from (**a**) control at 60 min or (**b**) pre-venom response to same agonist, *n* = 6.

*B. multicinctus* venom (1–10 μg/mL) caused concentration-dependent inhibition of indirect twitches in the chick biventer preparation when compared to vehicle (*n* = 6; one-way ANOVA, *p* < 0.05; Figure 1a). T_90_ values (i.e., time to cause 90% inhibition of twitches) for *B. multicinctus* venom were: 1 μg/mL, 35 ± 3 min; 3 μg/mL, 28 ± 2 min; and 10 μg/mL, 16 ± 1 min. *B. multicinctus* venom (1–10 μg/mL) abolished contractile responses to exogenous ACh (1 mM), CCh (20 μM), but not responses to KCl (40 mM), indicating an action at the post-synaptic nerve terminal but no myotoxicity (Figure 1b). 

#### 2.1.2. Prevention of In Vitro Neurotoxicity with Antivenom 

**Figure 2:**
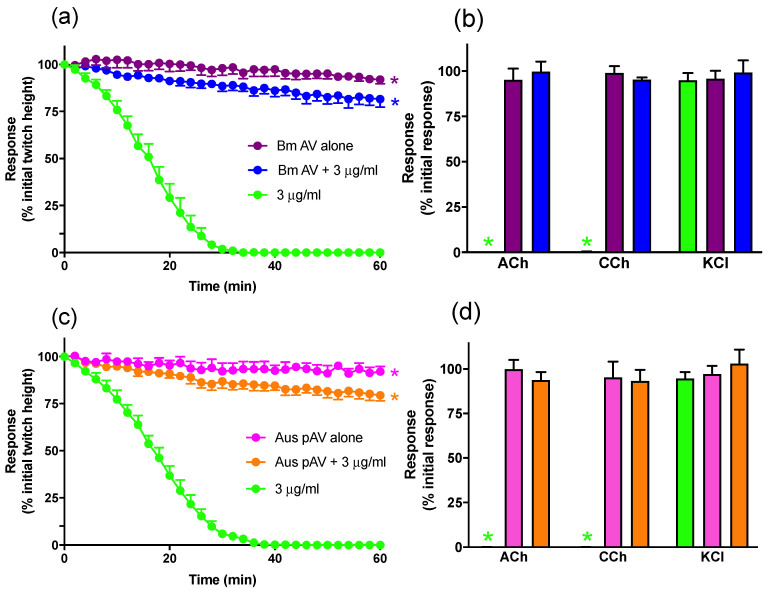
The effect of pre-addition of (**a**,**b**) Chinese *B. multicinctus* antivenom (Bm AV; 12 U/mL) or (**c**,**d**) Australian polyvalent antivenom (Aus pAV; 15 U/mL) on the neurotoxicity of *B. multicinctus* venom (3 μg/mL). Panels (**a**,**c**) show the effects on indirect twitches and panels (**b**,**d**) show the effects on responses to acetylcholine (ACh; 1 mM), carbachol (CCh; 20 μM) and potassium chloride (KCl; 40 mM), in the chick biventer cervicis nerve-muscle preparation. * *p* < 0.05, significantly different compared to venom in the absence of antivenom at 60 min (**a**,**c**) or compared to pre-venom response to same agonist (**b**,**d**). *n* = 5–6.

The prior addition of Chinese *B. multicinctus* antivenom (12 U/mL, 2× the recommended titer), or Australian polyvalent snake antivenom (15 U/mL, 50× the recommended titer), markedly attenuated the neurotoxic actions of *B. multicinctus* venom (3 μg/mL) (Figure 2a,c) and prevented the inhibition of contractile responses to ACh and CCh (Figure 2b,d). 

#### 2.1.3. Reversal of In Vitro Neurotoxicity with Antivenom and Washings

**Figure 3:**
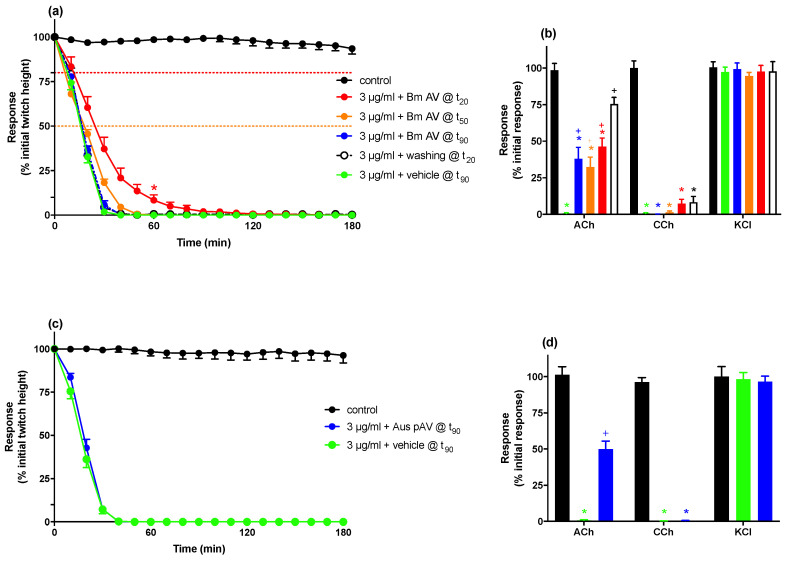
The effect of the addition of Chinese *B. multicinctus* antivenom (Bm AV; 60 U/mL) at the t_20_/t_50_/t_90_ time points or repeatedly washing at the t_20_, on *B. multicinctus* venom (3 μg/mL) neurotoxicity. Panel (**a**) shows the effects on indirect twitches and panel (**b**) shows the effects on responses to acetylcholine (ACh; 1 mM), carbachol (CCh; 20 μM) and potassium chloride (KCl; 40 mM), in the chick biventer cervicis nerve-muscle preparation. *n* = 6. The effect of the addition of Australian polyvalent antivenom (Aus pAV; 50 U/mL) at the t_90_ time point on *B. multicinctus* venom (3 μg/mL) neurotoxicity. Panel (**c**) shows the effects on indirect twitches and panel (**d**) shows the effects on responses to ACh (1 mM), CCh (20 μM) and KCl (40 mM), in the chick biventer cervicis nerve-muscle preparation. * *p* < 0.05, significantly different compared to venom alone at 60 min (**a**) or compared to pre-venom response to same agonist at 180 min (**b**,**d**). ^+^ *p* < 0.05, significantly different compared to response to agonist in the absence of antivenom or washing (**b**,**d**).

The addition of Chinese *B. multicinctus* antivenom (60 U/mL, 10× the recommended titer), at the t_90_ time point after the addition of *B. multicinctus* venom (3 μg/mL), did not restore twitch height (Figure 3a), but significantly reversed the venom-induced inhibition of responses to ACh (38 ± 8% of initial response) while having no significant effect on the response to CCh and KCl (Figure 3b).

The addition of Chinese *B. multicinctus* antivenom (60 U/mL, 10× the recommended titer), at the t_50_ or t_20_ time point after the addition of *B. multicinctus* venom (3 μg/mL), caused a significant delay in the time to abolish twitches (Figure 3a, Table 1), and significantly reversed the venom-induced inhibition of responses to ACh while having no significant effect on the responses to CCh or KCl (Figure 3b).

Repeatedly washing the tissue, commencing at the t_20_ time point after the addition of *B. multicinctus* venom (3 μg/mL), for 5 s every 1 min for 10 min, and then for 5 s every 5 min, did not produce any recovery of twitch height or delay in the time to abolish twitches (Figure 3a, Table 1) but did significantly reverse the venom-induced inhibition of responses to ACh (76 ± 4% of initial response). This had a minimal effect on the response to CCh, and no significant effect on the response to KCl (Figure 3b).

Comparison of the t_90_ values between venom alone, venom with Chinese *B. multicinctus* antivenom added at different time points, and venom with repeated washing, indicated that only antivenom addition at t_20_ significantly prolonged the t_90_ value compared to venom alone (*p* < 0.05, Table 1). There was no significant effect of washing commencing at t_20,_ or antivenom addition at t_50_ or t_90_.

The addition of Australian polyvalent antivenom (50 U/mL, 166× the recommended titer), at the t_90_ time point after the addition of *B. multicinctus* venom (3 μg/mL), did not restore the twitch height over a 180 min period (Figure 3c). However, the addition of Australian polyvalent antivenom significantly reversed the inhibition of responses to ACh while having no significant effect on the responses to CCh and KCl (Figure 3d).

## 3. Discussion

Chinese *B. multicinctus* venom caused concentration-dependent inhibition of nerve-mediated twitches in an in vitro skeletal muscle preparation. Contractile responses to nicotinic acetylcholine receptor agonists were attenuated indicating a postsynaptic site of action. Although it is worth noting that the effects of the potent, slower acting presynaptic neurotoxins, present in the venom, will be masked when examining whole venom. No evidence of myotoxicity was observed. The neurotoxic effects of the venom were almost completely abolished by the prior addition of *B. multicinctus* antivenom (at 2× the recommended titer) and an Australian polyvalent snake antivenom (50× the recommended titer). The latter is raised against a range of Australian elapid snakes (i.e., *Acanthophis antarcticus, Notechis scutatus, Oxyuranus scutellatus, Pseudechis australis,* and *Pseudonaja textilis*) but not *B. multicinctus* venom. Therefore, it seems as though both antivenoms recognize the post- and pre-synaptic neurotoxins found in *B. multicinctus* venom. This is not surprising as we have previously shown cross neutralization between Australian polyvalent snake antivenom and non-Australian snake species including the Chinese cobra, New Guinea small-eyed snake, monocled cobra, banded krait, common krait and king cobra [16,17,18]. The ability of other heterologous antivenoms to neutralize the neuromuscular effects of *Bungarus* genus venoms and toxins has also been reported previously [19,20,21].

In antivenom reversal studies, addition of either antivenom (i.e., Chinese *B. multicinctus* antivenom at 10× the recommended titer and Australian polyvalent antivenom at 166× the recommended titer), at the t_90_ time point after venom, was unable to reverse the abolition of twitches. To examine this further, we tried adding the antivenom at earlier time points (i.e., t_20_ and t_50_). While neither of these strategies prevented the abolition of twitches, responses to the nicotinic receptor agonist ACh were partially restored in all instances, indicating that the post-synaptic effects of the venom were at least partially reversible. Additionally, when antivenom was added at the t_20_ time point, there was a significant delay in the time taken to abolish twitches (based on a comparison of t_90_ values) indicating further reversal of the post-synaptic effects, and potentially pre-synaptic effects. In contrast, repetitive washing commencing at the same time point (i.e., t_20_) had no significant delaying effect on venom-induced neurotoxicity but did restore contractile responses to ACh indicating partial reversal of the post-synaptic activity of the venom but not presynaptic activity. 

As a comparison, we have recently shown that the in vitro post- and pre-synaptic neurotoxic effects of venom from the Australian coastal taipan (*Oxyuranus scutellatus*), in the same skeletal muscle preparation, can be delineated by the addition of antivenom at different time points after venom [22]. In this previous study, Australian polyvalent antivenom was able to prevent the pre-synaptic activity of *O. scutellatus* venom when added 5 min after the venom. However, antivenom was not effective when added 30 min after the venom and only partially effective when added 15 min after venom [22]. As a comparison, addition of antivenom at the t_20_ time point in the current study, equates to approximately 8 min after the venom. The lack of ability of antivenoms to reverse the effects of pre-synaptic β-neurotoxins is well established [23]. Given that *B. multicinctus* contains β-bungarotoxin, and the relatively irreversible long-chain α-bungarotoxin, it appears as though the ‘window’ for reversing the neurotoxic effects of *B. multicinctus* is very short. 

In vivo murine LD_50_ (i.e., the concentration of venom required to kill 50% of a population of mice, usually over a 24–48 h time period) studies are often used to determine venom lethality/toxicity, and antivenom neutralization activity. However, LD_50_ studies are unable to identify the toxins responsible for death and, if neurotoxins are involved, unable to identify the site of action of the neurotoxins given the fact that the venom may contain pre-, post-synaptic neurotoxins or both [14,24,25]. We used the chick biventer preparation, which contains both focally- and multiply-innervated skeletal muscle fibers, to examine *B. multicinctus* venom toxicity in vitro. This preparation enables the determination of the site of action of venoms/toxins, i.e., either at the pre-synaptic nerve terminal, post-synaptic nerve terminal or underlying skeletal muscle [24,26]. Pre-synaptic neurotoxins abolish nerve-evoked indirect twitches by interrupting release of acetylcholine from the nerve terminal [23,24]. While post-synaptic neurotoxins block the nerve-evoked twitches by blocking the interaction between the transmitter (i.e., acetylcholine) and the skeletal muscle nAChR [14,24]. These two different sites of action can be distinguished by examining the effects of the venom on responses to exogenous nAChR agonists. Pre-synaptic toxins do not affect responses, while post-synaptic toxins inhibit responses. However, when studying whole venoms, the presence of post-synaptic neurotoxins will mask the presence of pre-synaptic toxins given their faster and downstream site of action. Based on venomics data, *B. multicinctus* venom contains pre-synaptic and post-synaptic neurotoxins, but lacks myotoxic components [9,27,28]. This profile is likely to explain the lack of effect of the venom on contractile responses to KCl, which is used as a method of identifying toxins which directly affect the skeletal muscle at the end plate [26].

Clinical data from snakebites with primary pre-synaptic neurotoxins, such as kraits (*Bungarus* spp.) and taipans (*Oxyuranus* spp.) suggest that antivenom does not reverse established neurotoxicity, but early antivenom administration may be associated with decreased severity or even prevent neurotoxicity [25,29,30]. Post addition of Chinese *B. multicinctus* antivenom at earlier time points, i.e., t_20_ or t_50_, did delay the time to abolish twitches and significantly reversed the venom-induced inhibition of responses to ACh, indicating partial efficacy despite the onset of both ‘irreversible’ pre- and post-synaptic neurotoxicity. Interestingly, compared to t_20_ antivenom addition, repeated washing commencing at t_20_ did not delay the time to abolish twitches.

Therefore, the results of our in vitro studies, using the chick biventer cervicis preparation, mirror the clinical effects of *B. multicinctus* envenoming, where respiratory failure is a key feature in spite of early administration of the *B. multicinctus* antivenom [5]. This supports the hypothesis that the antivenom resistant neurotoxicity is most likely due to the action of the PLA_2_ β-bungarotoxins [10,11,12,13]. However, despite the close alignment of the experimental data with clinical outcomes, the extrapolation of data from experimental studies, including the chick biventer preparation, should be treated with caution given the species differences that have been reported, although these largely pertain to the activity of the post-synaptic α-neurotoxins [31,32,33].

A recent study examined the efficacy of *B. multicinctus* antivenom against isolated toxins (i.e., α-, β- and γ-bungarotoxins) from *B. multicinctus* venom using mice (i.e., an LD_50_ assay and determining ED_50_ values) [34]. These authors confirmed that β-bungarotoxin was the most ‘lethal’ component with LD_50_ values of 0.004 μg/g (i.p), 0.015 μg/g (i.m) and 0.007 μg/g (i.v.). Interestingly, an examination of the immunoreactivity of the antivenom, using ELISA, indicated that the antivenom showed good neutralizing capacity against the crude *B. multicinctus* venom but weak immunoreactivity against α-and γ-bungarotoxins [34]. In addition, the immunoreactivity of the antivenom was 2.5 times higher against the whole venom than β-bungarotoxin [34]. Therefore, even with early clinical administration, the antivenom is likely to display varying efficacy against key toxins.

## 4. Conclusions

We showed that Chinese *B. multicinctus* venom displays potent in vitro neurotoxicity which is attenuated by monovalent *B. multicinctus* antivenom and, also, an Australian polyvalent snake antivenom. Neither antivenom was able to reverse the venom-induced neurotoxicity. Given the seemingly irreversible nature of the neurotoxic effects of *B. multicinctus* venom, it would appear that early administration of the specific antivenom within a certain time window would be advantageous in the clinical setting.

## 5. Materials and Methods 

### 5.1. Venom and Antivenoms

Freeze-dried *B. multicinctus* venom (Orientoxin Co., Ltd., Shandong, China) was dissolved in 0.05% (*w*/*v*) bovine serum albumin and stored at −20 °C until required. Chinese *B. multicinctus* monovalent antivenom (Bm AV; Batch number: 2018/03/01; expiry date: 2021/03/19) was purchased from Shanghai Serum Biological Technology Co., Ltd. (Shanghai, China). Australian polyvalent snake antivenom (Aus pAV; Batch number: 055517501; expiry date: 04/2013) was purchased from Seqirus (Melbourne, Australia). According to the instructions from the manufacturer, 10,000 units of *B. multicinctus* neutralizes 5–6 mg of dried *B. multicinctus* venom. For the Australian polyvalent snake antivenom, 100 units of antivenom neutralizes 1 mg of dried venom from the species of snake against which the antivenom has been raised (i.e., brown snake, death adder, mulga snake, taipan, tiger snake). One vial of Aus pAV contains a total of 40,000 units. The amount of each antivenom required to neutralize the in vitro neurotoxic effects of the venom was calculated based on the relative amount of the venom in the organ bath.

### 5.2. Chemicals and Reagents

The following chemicals and drugs were used in the experiments described in this manuscript: acetylcholine chloride (ACh; Sigma-Aldrich, St. Louis, MO, USA), carbamylcholine chloride (CCh; Sigma-Aldrich, St. Louis, MO, USA), d-tubocurarine chloride (d-TC; Sigma-Aldrich, St. Louis, MO, USA), potassium chloride (KCl, Ajax Finechem Pty. Ltd., Taren Point, Australia), bovine serum albumin (Sigma-Aldrich, St. Louis, MO, USA). All chemicals were dissolved or diluted in Milli-Q water unless otherwise stated.

### 5.3. Chick Biventer Cervicis Nerve-Muscle Preparation

As previously described by us [15,16,18], male chickens (aged 4–10 days) were euthanized by exsanguination following CO_2_ inhalation. Biventer cervicis nerve-muscle preparations were dissected and then mounted on wire tissue holders under 1 g resting tension in 5 mL organ baths. Tissues were maintained at 34 °C, bubbled with 95% O_2_ and 5% CO_2_, in physiological salt solution (mM): 118.4 NaCl, 4.7 KCl, 1.2 MgSO_4_, 1.2 KH_2_PO_4_, 2.5 CaCl_2_, 25 NaHCO_3_ and 11.1 glucose. Indirect twitches (i.e., nerve-mediated) were evoked by stimulating the motor nerve (0.1 Hz; 0.2 ms) at supramaximal voltage (10–20 V), using a stimulator. Selective stimulation of the nerve was confirmed by the abolishment of twitches by the addition of d-TC (10 μM). Tissues were then repeatedly washed with physiological salt solution to restore twitch response to nerve stimulation. Contractile responses of the tissues to exogenous acetylcholine (ACh; 1 mM for 30 s), carbachol (CCh; 20 μM for 60 s) and KCl (40 mM for 30 s) were obtained in the absence of nerve stimulation. Nerve stimulation was then recommenced for at least 30 min before the addition of the venom or antivenom. To examine the ability of antivenom to neutralize venom induced neurotoxicity (i.e., neurotoxicity prevention study), tissues were equilibrated with antivenom for 10 min before venom was added. To determine the ability of antivenoms to reverse venom induced neurotoxicity (i.e., neurotoxicity reversal study), antivenom was added at t_90_ (i.e., time at which the initial twitch height was inhibited by 90%). The effect of earlier antivenom addition at t_20_ or t_50_ (i.e., time at which the initial twitch height was inhibited by 20% or 50%) was assessed for Bm AV. To further examine the reversibility of the neurotoxicity, when the twitch height had reduced to the t_20_ time point, the organ baths were repeatedly washed for 5 s every 1 min for 10 min and then washed for 5 s every 5 min until any recovery of twitches had plateaued or 3 h. At the conclusion of each experiment, ACh, CCh and KCl were re-added as above. Twitch responses and responses to exogenous agonists were measured via a Grass FT03 force displacement transducer and recorded on a PowerLab system (ADInstruments Pty Ltd., Bella Vista, NSW, Australia).

### 5.4. Data Analysis

As previously described by us [15,16,18], the height of electrically evoked twitches in the chick biventer cervicis preparation were measured at regular time intervals and expressed as a percentage of the pre-venom twitch height. The time taken for 90% inhibition of the twitch response (t_90_ values) was used to compare the neurotoxic effect of *B. multicinctus* venom following the addition of antivenom at different post-venom time points or repeat washing. Post-venom contractile responses to ACh, CCh and KCl were expressed as a percentage of their original responses. Comparison of the effects of *B. multicinctus* venom on twitch height was made using a one-way analysis of variance (ANOVA). Comparison of responses to exogenous agonists before and after the addition of *B. multicinctus* venom or vehicle was made using a Student’s paired *t*-test. All ANOVAs were followed by a Bonferroni’s multiple comparison post-hoc test. Data presented are in the form of mean ± standard error of the mean (SEM) of n experiments. All data and statistical analyses were performed using PRISM 8.0.2 (GraphPad Software, San Diego, CA, USA, 2019). For all statistical tests, *p* < 0.05 was considered statistically significant.

## Figures and Tables

**Table 1:** Comparison of t_90_ values for the neurotoxic effects of *B. multicinctus* venom in response to the addition of Chinese *B. multicinctus* antivenom or washing as indicated.

	Vehicle @ t_90_	Bm AV @ t_90_	Bm AV @ t_50_	Bm AV @ t_20_	Washing @ t_20_
t_90_ (min)	26.2 ± 0.9	28.0 ± 1.2	34.5 ± 1.3 ^†^	56.2 ± 8.0 *^,§^	26.0 ± 1.0 ^†^

Values are mean ± SEM, *n* = 6; * *p* < 0.05 significantly different from t_90_ (+vehicle) (one-way ANOVA). ^§^ *p* < 0.05 significantly different from Bm AV t_90_ addition (one-way ANOVA). ^†^ *p* < 0.05 significantly different from Bm AV t_20_ addition (unpaired *t*-test).
